# Photocontrol of Non-Adherent Cell Adhesion via Azobenzene–PEG–Lipid/Cyclodextrin Host–Guest Interactions

**DOI:** 10.3390/ijms27020562

**Published:** 2026-01-06

**Authors:** Masahiro Kawakami, Shinya Yamahira, Masaru Kojima, Satoshi Yamaguchi, Shinji Sakai

**Affiliations:** 1 Division of Chemical Engineering, Department of Materials Engineering Science, Graduate School of Engineering Science, The University of Osaka, 1-3 Machikaneyama-cho, Toyonaka-shi 560-8531, Osaka, Japan; kawamasa@cheng.es.osaka-u.ac.jp (M.K.);; 2 SANKEN (The Institute of Scientific and Industrial Research), The University of Osaka, 8-1 Mihogaoka, Ibaraki-shi 567-0047, Osaka, Japan

**Keywords:** host–guest interaction, PEG–lipid, photocontrol

## Abstract

Controlling cell attachment to substrates with spatiotemporal precision is a key technological foundation in fields such as tissue engineering, cell sorting, and cell–cell interaction analysis. Among existing approaches, azobenzene-based photocontrollable systems offer a promising strategy for the reversible regulation of cell adhesion. However, most conventional systems rely on the intrinsic adhesion capacity of adherent cells. Consequently, although the importance of non-adherent cell types has grown in biomedical research, their dynamic manipulation remains insufficiently explored. In this study, we developed a versatile system to control cell adhesion based on host–guest interactions between an azobenzene–lipid conjugate and a cyclodextrin-functionalized substrate. Using human chronic myelogenous leukemia (K562) cells, we successfully demonstrated photocontrolled adhesion and detachment, confirming the applicability of this system to non-adherent cells. Furthermore, we quantitatively measured the adhesion force and observed an inverse correlation between adhesion efficiency and adhesion force for different PEG linker lengths (2k, 4k, and 8k). This finding demonstrates the critical role of the linker length in effective cell surface modification. In conclusion, the proposed system establishes a photocontrollable adhesion method applicable to non-adherent cells, demonstrating its potential as a versatile technology for broad applications.

## 1. Introduction

Cells exhibit significant changes in their properties and behavior when they transition from an individual state to collective clusters [1,2]. Elucidating the interplay between individual cell behaviors and intercellular interactions is crucial for understanding fundamental biological phenomena such as wound healing [3,4], cancer metastasis [5,6], and immune responses [7,8,9]. Consequently, cell-patterning technologies have been actively investigated for over two decades to facilitate the precise positioning of cells and to observe their dynamic behavior.

Technologies for precise cell positioning can be broadly categorized into physical and chemical methods. Physical methods that utilize structures, such as microfluidic channels [10] or substrate topography [11], are effective for confining cell adhesion to specific areas. However, these methods often lack the flexibility required for dynamic and precise manipulations, such as altering prepatterned structures during cell behavior observations or selectively patterning multiple cell types.

To overcome this challenge, chemical methods that allow molecular-level control have been extensively explored [12,13,14,15,16,17]. These methods often combine chemical surface modification and microfabrication to achieve precise spatial control of cell adhesion. Approaches include the precise deposition of biomolecules via inkjet printing [12] and the creation of chemical patterns using light-sensitive materials or photocleavable surface linkers via photolithography [13]. Furthermore, soft lithography techniques such as microcontact printing enable the patterned transfer of specific chemical functionalities to substrates [14,15]. The application of these methods has enabled significant advances, including single-cell-resolution printing [15,16]. Molecular systems responsive to external stimuli, such as light, are particularly powerful tools for investigating cell–cell communication and evaluating drug responses, as they allow the spatiotemporal on/off control of cell adhesion [17]. For instance, the 2-nitrobenzyl group, which can be cleaved upon UV irradiation, has been widely used to analyze cell migration [3,4] and to selectively alter substrate properties to observe the heterogeneity of cytotoxic interactions between single immune cells and cancer cells [7]. Although photocleavage-based methods are powerful, their irreversible nature limits their utility for dynamic manipulation.

Dynamic control systems based on photoisomerization of azobenzene have been developed to achieve reversibility. This property has been leveraged to regulate cell adhesion by controlling the accessibility of peptides such as Arg-Gly-Asp (RGD), to cells [18,19,20,21]. Furthermore, azobenzene forms a specific host–guest complex with cyclodextrin, a cyclic oligosaccharide [22]. This photoresponsive interaction is biocompatible and easy to implement, enabling applications in drug delivery systems [23], cell–cell adhesion control [24,25], and dynamic scaffolding materials [26]. Although reversible cell adhesion systems based on this host–guest interaction have been reported, they typically rely on cell-specific targeting moieties, such as RGD peptides [8,27] and aptamers [28]. Consequently, the reliance on such specific interactions restricts the applicability of these systems, primarily to adherent cells. This limitation excludes non-adherent cell populations, such as immune cells, hematological cancer cells, circulating tumor cells, and certain stem cell subclasses, for which analyzing and controlling cell–cell interactions has become increasingly vital in recent years [29]. Therefore, applying this azobenzene-based strategy to non-adherent cells is essential for establishing a versatile method for dynamic cell adhesion control.

Although materials based on PEG and azobenzene have been extensively studied for applications such as self-assembly, drug delivery, and hydrogels [30,31,32,33], their application in cell membrane engineering for regulating cell adhesion has not been explored. To address this gap, this study aimed to develop a molecular system that enables photocontrol of non-adherent cell adhesion and detachment. We synthesized azobenzene–BAM (AzoBAM) by conjugating azobenzene to a biocompatible anchor for cell membrane (BAM), containing an oleyl group for direct receptor-independent insertion into the lipid bilayer [34]. We then prepared a corresponding substrate by immobilizing β-cyclodextrin (β-CD) on a glass surface (CD-glass). In this system, cell adhesion is mediated by the host–guest complexation between the trans-Azo moiety on the cell membrane and the β-CD on the substrate. Herein, we report the synthesis and characterization of these molecules and demonstrate their application in photocontrolled cell manipulation using human chronic myelogenous leukemia (K562) cells as a non-adherent model. Furthermore, we investigated the relationship between adhesion efficiency and adhesion force to optimize the molecular design. This study provides a novel strategy for manipulating non-adherent cells by targeting the ubiquitous lipid bilayer, offering a distinct approach compared to conventional PEG–azobenzene materials.

## 2. Results and Discussion

### 2.1. Surface Modification and Characterization

**Figure 1:**
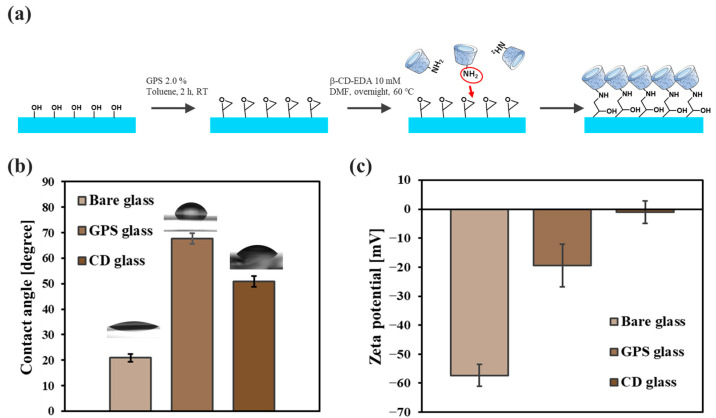
(**a**) Schematic of the glass surface modification process. The arrow indicates the nucleophilic attack of the amino group of the β-cyclodextrin (β-CD) derivative on the epoxy group of 3-glycidyloxypropyltrimethoxysilane (GPS). (**b**) Water contact angles of bare glass, GPS-modified glass, and β-CD-modified glass (CD-glass). (**c**) Surface zeta potentials of bare glass, GPS-modified glass, and CD-glass. Error bars represent standard error (*n* = 3).

The surface-modification strategy employed to prepare the CD-glass substrates is shown in Figure 1a. The stepwise functionalization of the glass surface was verified by water contact angle measurements and surface zeta potential analysis. As shown in Figure 1b, the introduction of epoxy groups via the 3-glycidyloxypropyltrimethoxysilane (GPS) treatment significantly increases the water contact angle relative to that of bare glass, indicating a decrease in surface hydrophilicity. The subsequent immobilization of the hydrophilic β-CD moieties, selected to prevent nonspecific inclusion of the PEG linker due to cavity size mismatch [35], restores the wettability, resulting in a reduced contact angle.

Furthermore, the surface zeta potential gradually shifts towards neutrality with each modification step (Figure 1c). The bare glass exhibits a highly negative potential because of the presence of deprotonated silanol groups (–Si–O^−^). Functionalization with GPS masks these negative charges by introducing neutral epoxide groups, causing a significant positive shift. The subsequent grafting of β-CD, which is also electrically neutral, further shifts the potential towards 0 mV. This suggests that the β-CD molecules physically shield the remaining silanol groups that are not fully covered by the initial GPS layer, thereby further neutralizing the surface potential. Collectively, these physicochemical changes demonstrate the successful construction of the CD-glass substrates.

### 2.2. Synthesis and Photoresponsive Properties of AzoBAM

**Figure 2:**
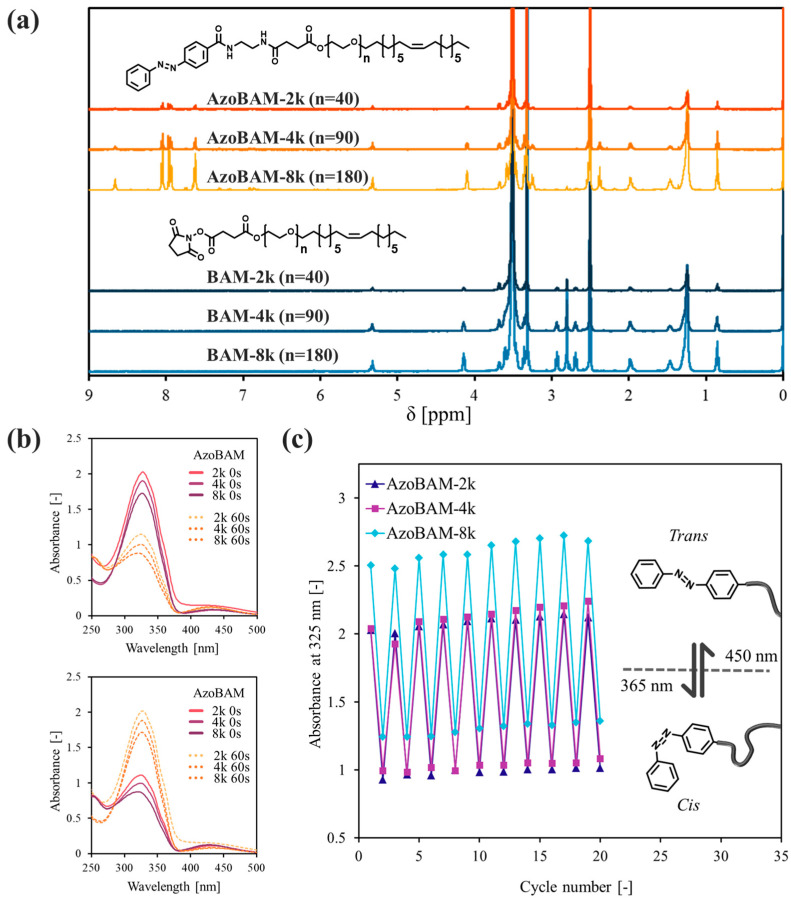
(**a**) ^1^H NMR spectra of biocompatible anchor for cell membrane (BAM) and azobenzene–BAM (AzoBAM) variants (2k, 4k, and 8k). (**b**) UV-vis absorbance spectra showing the photoisomerization of AzoBAM. The top graph shows the *trans*-to-*cis* transition under 365 nm irradiation (16.6 W/m^2^), and the bottom graph shows the *cis*-to-*trans* transition under 450 nm irradiation (157 W/m^2^). In both panels, solid and dashed lines indicate spectra before (0 s) and after (60 s) irradiation, respectively. (**c**) Cyclic photoisomerization monitored by changes in absorbance at 325 nm during alternating irradiation with 365 and 450 nm light.

**Scheme 1:**
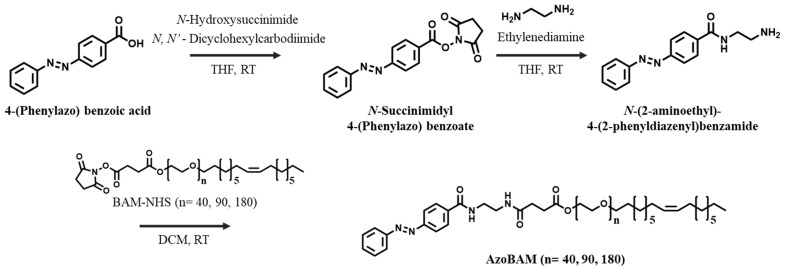
Synthesis of AzoBAM.

The AzoBAM linkers were synthesized using a two-step conjugation strategy (Scheme 1). First, 4-(phenylazo)benzoic acid was derivatized with ethylenediamine to introduce a reactive amine. This amine-functionalized Azo was then conjugated to BAM-NHS to yield the final AzoBAM variants with molecular weights of 2k, 4k, and 8k. The chemical structures were verified by ^1^H NMR spectroscopy; specifically, the characteristic signals in the 7.5–8.2 ppm region, assigned to the aromatic protons of the Azo moiety, verify successful conjugation (Figure 2a).

The photoresponsive behavior of AzoBAM was evaluated by UV-vis spectroscopy. Upon irradiation with 365 nm light, the strong absorption band of the *trans*-isomer at 325 nm decreases significantly, accompanied by a concurrent increase in the *cis*-isomer band at 429 nm. This *trans*-to-*cis* transition reached a photostationary state within 60 s (Figure 2b). The process was fully reversible; subsequent irradiation with 450 nm light induced complete reversion to the *trans* state within 60 s (Figure 2b). To demonstrate the robust repeatability, the solution was subjected to alternating cycles of irradiation at 365 and 450 nm. The absorbance at 325 nm confirms that AzoBAM undergoes repeated, reversible photoisomerization (Figure 2c). Although a slight increase in absorbance intensity was observed over the cycles, this was attributed to an increase in concentration caused by solvent evaporation during prolonged measurements, as the spectral shape remained unchanged (Figure S1b).

### 2.3. Cell Adhesion Control via Host–Guest Interaction

**Figure 3:**
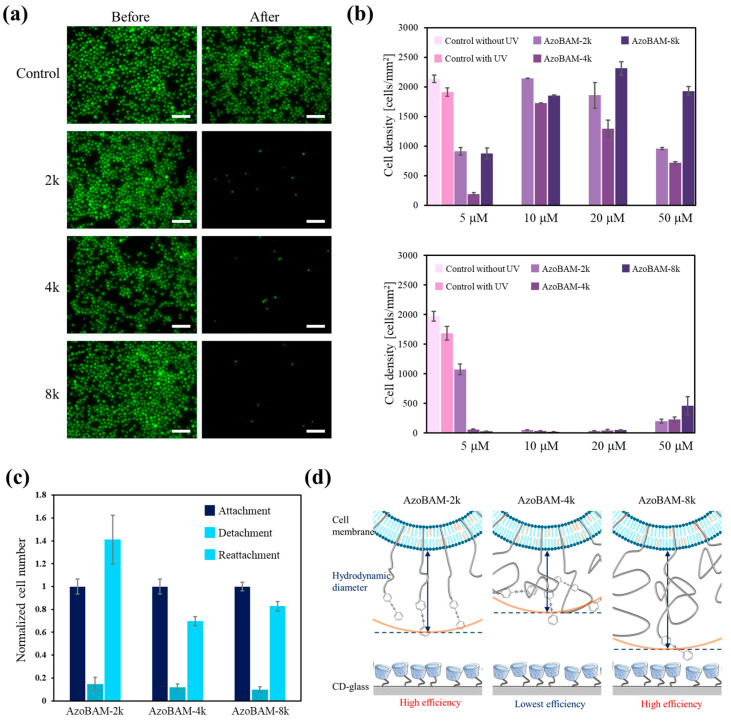
(**a**) Fluorescence images demonstrating photocontrolled cell adhesion. Representative images of unmodified control cells and cells modified with AzoBAM (10 µM). Scale bars; 100 µm. (**b**) Cell adhesion efficiency at various AzoBAM concentrations. Top and bottom panels show the cell density before and after UV irradiation (365 nm), respectively. (**c**) Quantitative analysis of the number of adhered cells during the attachment, detachment, and reattachment cycle. Experiments were performed at an AzoBAM concentration of 10 µM, and the number of adhered cells was normalized to the initial attachment value. Error bars represent standard error (*n* = 5). (**d**) Schematic of the molecular spreading of AzoBAM variants on the cell membrane. The highly compact 4k variant exhibits the lowest adhesion efficiency because of its limited physical reach to the substrate. The arrow indicates the hydrodynamic diameter.

First, we evaluated the nonspecific adhesion of cells lacking AzoBAM modification to CD-glass as a negative control. This experiment aimed to confirm that subsequent photocontrol was dependent on AzoBAM. Unexpectedly, the unmodified cells exhibited significant nonspecific adhesion. Given that CD forms inclusion complexes with various biomolecules, such as lipids and proteins [36], it is plausible that this nonspecific adhesion results from interactions with endogenous molecules present on the cell surface [37]. Subsequently, we investigated the effect of UV light on nonspecific adhesion by comparing cell retention with and without UV irradiation (Figure 3a). The non-irradiated control retains 92.4% of its initial cell density after washing, indicating a minor loss due to physical shear stress. In comparison, the UV-irradiated sample (365 nm) exhibits 88.2% retention (Figure 3b). Although UV irradiation results in slightly lower retention, the difference is marginal. We also confirmed that the applied irradiation (1 min) had no significant effect on cell viability after 24 h (Figure S2a,b).

Although we confirmed the cytocompatibility of short-term UV irradiation in this study, the use of visible or near-infrared (NIR) light would be advantageous for broader biological applications. Visible and NIR light offer deeper tissue penetration and further reduced phototoxicity compared to UV light. Recent studies have reported successful photocontrol using these longer wavelengths with azobenzene derivatives [38,39,40,41]. Incorporating such visible- or NIR-responsive moieties into the AzoBAM design represents a promising direction for future research.

Next, the specific adhesion behavior of the cells modified with different AzoBAM variants was investigated (Figure S3). For AzoBAM-2k, optimal adhesion efficiency is observed at a modification concentration of 10 µM, where subsequent UV irradiation triggers the detachment of most cells. In contrast, at a lower concentration of 5 µM, cells adhered but failed to detach upon UV irradiation (Figure 3b). This suggests that, at this low concentration, the short molecular chain and sparse distribution of AzoBAM-2k are insufficient to overcome the baseline nonspecific adhesion forces mediated by native cell surface components. The longer-chain variants, AzoBAM-4k and -8k, demonstrate maximal adhesion efficiencies at 10 and 20 µM, respectively, with successful UV-induced detachment across all tested concentrations. However, the overall adhesion efficiency of AzoBAM-4k is consistently lower than those of the 2k and 8k variants. To evaluate the reversibility of the system, we performed an attachment–detachment–reattachment cycle using the optimal AzoBAM concentration (10 µM). As shown in Figure 3c, high re-adhesion efficiency was observed, demonstrating that AzoBAM-mediated cell adhesion can be controlled in a reversible manner.

To explain this counterintuitive observation regarding the adhesion efficiency, we propose a hypothesis based on a distinct governing mechanism. Cell adhesion initiation requires the terminal Azo moieties to physically contact and form inclusion complexes with CD molecules on the substrate. Ligand binding via tethered polymer chains is governed by the probability that the polymer chain adopts an extended conformation to bridge the gap between the surfaces [42]. Consequently, a compact conformation hinders bond formation, whereas an extended conformation facilitates it. In this context, a previous study that modified BAM molecules on iron nanoparticle surfaces reported a nonlinear trend in the hydrodynamic diameter at low grafting densities: 8k > 2k > 4k [43]. To support this view, our isothermal titration calorimetry (ITC) results indicated that the 4k variant exhibited a higher tendency for molecular aggregation or compaction in solution compared to the 8k variant (Figure S4). This finding aligns with the trend reported in reference [43], reinforcing the hypothesis that AzoBAM-4k adopts a collapsed conformation on the cell membrane. This structural compactness significantly suppresses the probability of the spontaneous chain extension required to bridge the gap with the substrate. Consequently, the reduced probability of the terminal Azo reaching the substrate results in the lowest adhesion efficiency observed for AzoBAM-4k (Figure 3d).

### 2.4. Single-Cell Adhesion Force Measurement

**Figure 4:**
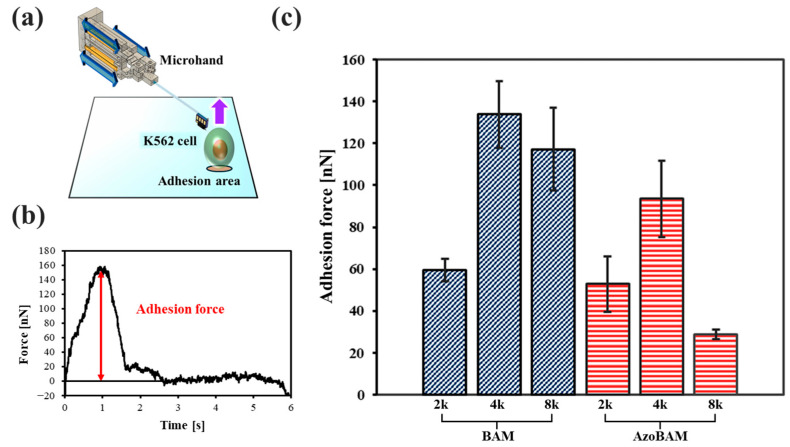
(**a**) Schematic of single-cell adhesion force measurement using a microhand system. The needle-shaped end-effector was inserted into the cell and retracted vertically. (**b**) Representative force-time curve obtained during the measurement. The maximum peak indicates the adhesion force, indicated by the red arrow. (**c**) Adhesion forces for K562 cells in the BAM system (dark blue cross-hatched bars) and the AzoBAM system (orange horizontally striped bars). In each group, data for PEG linker lengths of 2k, 4k, and 8k are shown from left to right. Error bars represent standard error (*n* ≥ 12 for BAM, *n* ≥ 5 for AzoBAM).

To quantify and compare cellular adhesion forces, we utilized a microhand system to analyze two distinct adhesion modalities (Figure 4a,b): the photocontrollable AzoBAM system, where adhesion occurs between AzoBAM-modified cells and a CD-glass substrate; and the BAM control system, comprising unmodified cells on a BAM-functionalized substrate, where binding is mediated by hydrophobic oleyl group insertion into the cell membrane (Figure 4c).

First, to validate the measurement system, we evaluated the BAM-2k variant. The measured adhesion forces (59.5 ± 5.4 nN, *n* = 15) are highly consistent with previously reported values (~60–80 nN) under similar conditions [44], confirming the reliability of our setup. The adhesion forces across the entire control BAM series were compared. The results revealed a clear trend in which adhesion force follows the order BAM-4k (133.8 ± 15.9 nN, *n* = 12) > BAM-8k (117.2 ± 19.6 nN, *n* = 17) > BAM-2k.

Next, we measured the adhesion force of the photocontrollable AzoBAM system using cells treated with 10 µM of each variant. Consistent with the BAM control system, AzoBAM-4k exhibits the highest adhesion force (93.5 ± 18.2 nN, *n* = 5). However, in contrast to the control trend, AzoBAM-8k shows the lowest force (28.7 ± 2.4 nN, *n* = 6), while AzoBAM-2k shows an intermediate value (52.9 ± 13.2 nN, *n* = 7).

In contrast to the factors governing initial cell capture (adhesion efficiency), discussed in Section 2.3, the adhesion force, defined as the strength of the established interface, shows a markedly different dependence on the linker length. Although the adhesion mechanisms differ between the two systems, analysis of the relative reduction in force compared with the BAM control provides critical insights into the effect of the PEG linker. We observe a molecular weight-dependent decline in force retention: ~11% for the 2k variant, ~30% for the 4k variant, and ~75% for the 8k variant. This suggests that steric hindrance is the dominant factor affecting the longer chains. For AzoBAM-8k, the substantial steric bulk of the PEG chain likely physically obstructs the terminal Azo from accessing the CD cavities, resulting in a severe loss of binding efficiency. Conversely, for AzoBAM-2k, the reduction was minimal, indicating that the short chain imposed a minor steric penalty. However, the absolute adhesion force remained low, reflecting the inherently weak membrane anchorage of the short BAM-2k moiety. Consequently, AzoBAM-4k was identified as the optimal design, with a PEG chain short enough to minimize steric hindrance and long enough to maintain robust adhesion.

Integrating the results of the adhesion efficiency (Figure 3b) and adhesion force (Figure 4c) reveals a striking inverse correlation across different AzoBAM molecular weights. Although the 4k variant exhibits the lowest adhesion efficiency owing to its compact conformation, which limits its initial reach, it paradoxically provides the highest adhesion force by balancing the reach and steric hindrance. Conversely, the 2k and 8k variants exhibit adhesion higher efficiencies, but significantly lower forces. We attribute this counterintuitive result to the complex interplay between the PEG linker lengths, which indicates a critical tradeoff between molecular extension and steric hindrance.

## 3. Materials and Methods

### 3.1. Materials

SUNBRIGHT^®^ OE-020CS, OE-040CS, and OE-080CS (BAM-NHS 2k, 4k, and 8k with molecular weights of 2144, 4237, and 8235, respectively) were purchased from NOF Corporation (Tokyo, Japan). 3-Glycidyloxypropyltrimethoxysilane was purchased from Sigma-Aldrich (St. Louis, MO, USA). Bovine serum albumin (BSA, fatty acid-free) was purchased from FUJIFILM Wako Pure Chemical (Osaka, Japan). K562 cells were obtained from RIKEN BioResource Research Center (Tsukuba, Japan). RPMI 1640 medium and fetal bovine serum (FBS) were purchased from Nissui Pharmaceutical (Tokyo, Japan) and Gibco (Grand Island, NY, USA), respectively. Calcein-AM was purchased from Nacalai Tesque Inc. (Kyoto, Japan). Information regarding the other reagents used for synthesis is provided in the Supplementary Materials.

### 3.2. General Measurements

^1^H NMR spectra were recorded on a JNM-ECS400 (400 MHz) spectrometer (JEOL Ltd., Tokyo, Japan) at 20 °C. The surface zeta potentials of the modified substrates were measured at room temperature using a zeta potential analyzer (ELSZ-2000, Otsuka Electronics, Osaka, Japan). UV-vis absorbance spectra were recorded using a UV-2600 spectrometer (Shimadzu Corporation, Kyoto, Japan). To induce photoisomerization, UV-LEDs (16.6 W/m^2^, EX-365/L; Optocode Corporation, Tokyo, Japan) and visible LEDs (157 W/m^2^, EX-450; Optocode Corporation) were used. Light intensities were measured using CL-70F (Konica Minolta Japan Inc., Tokyo, Japan) and USR-45 (Ushio Inc., Tokyo, Japan). Water contact angles were measured using a contact angle meter (LSE-ME3, Nick Corporation, Saitama, Japan). A droplet of deionized (DI) water (1 µL) was gently deposited onto the sample surface at room temperature, and the contact angle was measured immediately. While quantitative purity analysis was not performed, careful purification protocols were employed for all synthesized compounds. ^1^H NMR spectroscopy was used to confirm the structural identity and the absence of significant impurities within the detection limits.

### 3.3. Preparation of CD-Functionalized Glass Substrates (CD-Glass)

Mono-6-(2-aminoethyl)amino-6-deoxy-β-CD (β-CD-EDA) was synthesized according to the reported procedure [45], and the detailed protocol is provided in the Supplementary Materials (Figure S5). Borosilicate glass substrates were cleaned by sonication in water and ethanol. The substrates were immersed in a 1 M aqueous KOH solution for 4 h. After rinsing with DI water and drying under vacuum at room temperature, the substrates were allowed to react with a GPS solution in anhydrous toluene (2.0% *v*/*v*) for 2 h at room temperature. The substrates were subsequently washed with toluene and ethanol and cured for 15 min at 120 °C. Finally, the GPS-modified glass substrates were immersed in a solution of β-CD-EDA (10 mM in DMF) and incubated overnight at 60 °C to obtain the final CD-glass (Figure 1a).

### 3.4. Synthesis of AzoBAM Variants (2k, 4k, 8k)

The synthetic route for AzoBAM is illustrated in Scheme 1 and detailed synthesis procedures are provided in the Supplementary Materials (Figure S6). The precursors, *N*-succinimidyl 4-(phenylazo)benzoate and *N*-(2-aminoethyl)-4-(2-phenyldiazenyl)benzamide, were prepared according to previously reported methods [46,47]. BAM-NHS (200.8 mg for 2k, 203.3 mg for 4k, and 202.3 mg for 8k) were dissolved in 10 mL of anhydrous THF. To each solution, *N*-(2-aminoethyl)-4-(2-phenyldiazenyl)benzamide (30.3, 15.5, and 8.1 mg for 2k, 4k, and 8k BAM, respectively; 1.2 equiv.) was added, and the resulting mixture was stirred overnight at room temperature. The mixtures were diluted with DCM (10 mL) and washed sequentially with a saturated aqueous NaHCO_3_ solution and brine. The organic phases were dried over anhydrous Na_2_SO_4_, filtered, and then concentrated under reduced pressure. The resulting solids were dissolved in anhydrous DCM (2 mL) and purified by precipitation in cold diethyl ether (50 mL, 0 °C). The resulting precipitates were collected by filtration and dried under vacuum to yield the final AzoBAM products as orange solids (Yields: AzoBAM-2k: 136.4 mg, 63%; 4k: 129.2 mg, 61%; 8k: 135.4 mg, 65%).

### 3.5. Photoisomerization of AzoBAM

Working solutions (100 µM) of AzoBAM variants (2k, 4k, and 8k) were prepared by diluting a 10 mM stock solution (in DMSO) with PBS. An aliquot (200 µL) of each solution was used for photoisomerization analysis. First, the time required to achieve complete *trans*-to-*cis* isomerization (365 nm irradiation) and *cis*-to-*trans* isomerization (450 nm irradiation) was determined by monitoring the changes in the absorbance spectra (Figure S1a). Subsequently, to evaluate the stability of the reversible isomerization, the solution was subjected to alternating irradiation cycles at 365 and 450 nm, with 90 s irradiation time per step to ensure complete photoswitching.

### 3.6. Cell Culture and AzoBAM Modification

K562 cells were maintained in RPMI 1640 medium supplemented with 10% FBS, 2 mM L-glutamine, and 100 U/mL streptomycin in a humidified incubator at 37 °C with 5% CO_2_. For surface modification, cells were harvested and resuspended in PBS. AzoBAM stock solutions (10 mM in DMSO) were added to the cell suspension to achieve final concentrations of 5, 10, 20, or 50 µM. The mixture was incubated at 37 °C for 5 min to allow membrane insertion. After incubation, the cells were washed twice with PBS to remove excess AzoBAM molecules.

### 3.7. Single-Cell Adhesion Force Measurements

The cell adhesion force was measured using a microhand micromanipulation system, as previously described [48]. To prevent nonspecific cell adhesion to the end-effector, the end-effector tips were treated with 1.0% (*w*/*v*) BSA solution for 1 h before measurement. For the measurement, needle-shaped end-effector tips were inserted into a single cell that adhered to the substrate. The cells were then retracted vertically (Figure 4a), and the adhesion force was determined from the maximum reaction force (Figure 4b).

### 3.8. Evaluation of Photocontrolled Cell Adhesion

K562 cells were harvested by centrifugation (1800 rpm, 2 min; CT15RE T15A61; Eppendorf Himac Technologies, Tokyo, Japan) and washed three times with PBS. For visualization, the cells were stained with Calcein-AM. In preliminary experiments, propidium iodide (PI) staining was performed to assess membrane integrity after AzoBAM treatment, and no apparent increase in PI-positive cells was observed under the experimental conditions used (Figure S2c). A stock solution of Calcein-AM (1 mg/mL in DMSO) was diluted 500-fold with PBS, and the cells were incubated at 37 °C for 30 min. After washing with PBS, the cells were modified with AzoBAM, according to the procedure described in Section 3.6. Following the modification, the cells were washed twice with PBS and resuspended at a concentration of 5.0 × 10^5^ cells/mL. The cell suspension was seeded onto a CD-glass substrate and incubated at 37 °C for 30 min. After washing thrice with PBS, the cells were observed under a fluorescence microscope (BZ-X800L, Keyence Co., Osaka, Japan). To induce cell detachment, the substrate was irradiated with 365 nm light for 1 min. The substrate was then washed three times with PBS, and the remaining cells were visualized to assess detachment efficiency. To evaluate the reusability, a reattachment experiment was subsequently performed. Freshly prepared AzoBAM-modified K562 cells were seeded onto the same CD-glass substrate following the identical procedure described above. After incubation at 37 °C for 30 min, the substrate was washed three times with PBS. Adherent cells were visualized by fluorescence microscopy with using a 10× objective lens. The number of cells in five distinct fields of view was manually counted using the ImageJ software (version 1.54g). The average cell density was determined by normalizing the cell counts to the image area.

### 3.9. Preparation of BAM-Functionalized Control Substrates

The glass substrates were first coated by immersion them in a 1.0% (*w*/*v*) BSA solution overnight at room temperature. Working solutions (100 µM) of BAM-NHS (2k, 4k, and 8k) were prepared by diluting 10 mM stock solutions (in DMSO) with PBS. The BSA-coated substrates were rinsed with PBS and subsequently incubated in the BAM-NHS solution for 1 h at 37 °C. In this step, the *N*-hydroxysuccinimide (NHS) ester of BAM reacts with the amino groups of the BSA layer, covalently immobilizing lipid anchors on the surface. After the reaction, the substrates were washed five times with PBS to remove the unreacted reagents and mounted on the stage of the microhand system. The K562 cells were harvested, washed, and resuspended in PBS. The cell suspension was then applied to the BAM-functionalized surface and incubated for 20 min at room temperature to allow cell adhesion. Single-cell adhesion forces were then measured as described in Section 3.7.

## 4. Conclusions

In this study, we developed a photocontrol system for cell adhesion by combining a novel molecule, AzoBAM (2k, 4k, and 8k), with a β-CD-functionalized glass substrate. Using K562 cells, we demonstrated efficient capture and subsequent UV-induced detachment. This result validated the applicability of this lipid bilayer-targeting strategy to non-adherent cells, highlighting its potential as a versatile platform for dynamic cell manipulation. In addition, this study identified a critical tradeoff in the PEG linker design for the AzoBAM system. The optimal 4k linker length highlights the balance between maintaining stable membrane anchorage and minimizing steric hindrance. This insight, which demonstrates that macroscale adhesion efficiency and microscale adhesion force do not necessarily correlate, provides crucial guidance for the rational design of future cell surface modifiers.

## Data Availability

The original contributions presented in this study are included in the article/Supplementary Materials. Further inquiries can be directed to the corresponding author.

## Supplementary Materials

The following supporting information can be downloaded at: https://www.mdpi.com/article/10.3390/ijms27020562/s1.

## Abbreviations

The following abbreviations are used in this manuscript:

PEG	Polyethylene glycol
DMF	*N*,*N*-dimethylformamide
THF	Tetrahydrofuran
DCM	Dichloromethane
DMSO	Dimethyl sulfoxide
PBS	Phosphate-buffered saline
