# Supplementary material for: Photocontrol of Non-Adherent Cell Adhesion via Azobenzene–PEG–Lipid/Cyclodextrin Host–Guest Interactions
Source: Int J Mol Sci. 2026 Jan 6;27(2):562. doi: 10.3390/ijms27020562 (PMC12840613; doi:10.3390/ijms27020562)
Supplement: Supplementary file 1 [file ijms-27-00562-s001.zip › ijms-4063515-supplementary.pdf]

Supplementary Materials

Photocontrol of Non-adherent Cell Adhesion via Azobenzene-PEG-Lipid/Cyclodextrin Host-Guest Interactions

Masahiro Kawakami [1], Shinya Yamahira [2], Masaru Kojima [1,*], Satoshi Yamaguchi [2] and Shinji Sakai [1]

1. General Information

4-(Phenylazo)benzoic acid and *p*-toluenesulfonyl chloride (TsCl) were purchased from Tokyo Chemical Industry (Tokyo, Japan). β-Cyclodextrin (β-CD) was purchased from Junsei Chemical (Tokyo, Japan). Ethylenediamine (EDA), *N*-hydroxysuccinimide (NHS), and *N,N′*-dicyclohexylcarbodiimide (DCC) were of reagent grade and purchased from FUJIFILM Wako Pure Chemical (Osaka, Japan). All the reagents were used without further purification.

^{1}H NMR spectra were recorded on a JEOL JNM-ECS400 (400 MHz) spectrometer at 20 °C. Automated flash column chromatography was performed using a Biotage® Selekt Enkel system (Biotage Japan Ltd., Tokyo, Japan).

2. Methods

2.1. Synthesis of Mono-6-tosyl-β-cyclodextrin (β-CD-OTs)

β-CD (10.1 g, 8.90 mmol) was dissolved in DI water (80 mL) in a flask. A solution of NaOH (4 M, 6.84 mL) was added dropwise to the β-CD solution over 10 min while stirring in an ice-water bath. Subsequently, a solution of TsCl (1.99 g, 10.4 mmol) was added dropwise in acetonitrile (5.0 mL) over 1 h at the same temperature. The reaction mixture was then stirred at room temperature for 4 h. The resulting precipitate was removed by vacuum filtration, and the pH was adjusted to ~6.0, using 1 M HCl. The solution was stored at 4 °C overnight to induce recrystallization. The white crystalline product was collected by vacuum filtration, washed three times with cold acetone, and dried under vacuum to yield the title compound (1.70 g, 15%). ^{1}H NMR (400 MHz, DMSO-d_6): δ 7.75 (d, *J* = 7.3 Hz, 2H, Ar-H), 7.43 (d, *J* = 7.7 Hz, 2H, Ar-H), 5.71–5.64 (m, 14H, OH-2,3), 4.83 (d, *J* = 3.5 Hz, 7H, H-1), 4.52–4.38 (m, 6H, OH-6 and H-6'), 3.64–3.40 (m, 28H, H-3,5,6), 3.40–3.30 (m, 14H, H-2,4 overlapped with HDO), 2.43 (s, 2.5H, Tosyl-CH_3) ppm (Figure S1 (b)). The degree of substitution (DS) was calculated to be 0.80 based on the integral ratio of the tosyl aromatic signals to the anomeric signal (H-1), confirming the successful synthesis of mono-tosylated β-CD.

2.2. Synthesis of Mono-6-(2-aminoethyl)amino-6-deoxy-β-cyclodextrin (β-CD-EDA)

β-CD-EDA was synthesized according to the reported procedure [1], as illustrated in Figure S1(a). β-CD-OTs (1.0 g, 0.79 mmol) were dissolved in EDA (10 mL), and the solution was stirred at 80 °C for 4 h under a nitrogen atmosphere. After the reaction was complete, excess EDA was removed by rotary evaporation. The crude residue was dissolved in a minimum amount of a methanol/water mixture (1:1, v/v) and precipitated by adding the solution dropwise to a large volume of cold acetone with vigorous stirring. This dissolution–precipitation cycle was repeated three more times for purification. The final white product was collected by filtration and dried under vacuum (889.4 mg, 96%). ^{1}H NMR (400 MHz, D_2O): δ 5.09 (d, *J* = 3.5 Hz, 7H, H-1), 4.82 (HDO), 3.99–3.86 (m, 28H, H-3,5,6), 3.68–3.58 (m, 14H, H-2,4), 2.91 (m, 4H, EDA-CH_2) ppm (Figure S1 (c)). The DS of the amino group was determined to be **0.9** by ^{1}H NMR integration ratios. Since unreacted β-CD lacks the amino group and does not participate in the subsequent conjugation reaction, the mixture was used without further separation of the mono-substituted product.

2.3. Synthesis of N-succinimidyl 4-(phenylazo)benzoate

Although the preparation of the corresponding substance has been reported previously [2], we synthesized the target NHS ester based on the reported method with a modified purification procedure. NHS (368.8 mg, 3.20 mmol) was added to a solution of 4-(phenylazo) benzoic acid (601 mg, 2 mmol) in THF (20 mL), and the mixture was stirred. Subsequently, DCC (659.9 mg, 3.20 mmol) was added and the mixture was stirred overnight at room temperature. After the reaction, dichloromethane (DCM, 20 mL) was added and the solution was washed sequentially with DI water (40 mL), saturated aqueous $NaHCO_3$, and saturated aqueous NaCl (brine). The organic phase was dried over anhydrous Na_2SO_4, filtered, and concentrated under a vacuum.

The crude product was purified by column chromatography using ethyl acetate and hexane as solvents. After chromatography, the solvent was evaporated, and the resulting orange powder was dissolved in ethyl acetate (100 mL) and stored at 4 °C overnight. The white precipitate formed was removed by filtration. Finally, the solvent was evaporated from the filtrate to obtain *N*-succinimidyl-4-(phenylazo)benzoate as an orange powder (653.3 mg, 76% yield). ^{1}H NMR (400 MHz, DMSO-d_6): δ 8.33 (d, *J* = 8.8 Hz, 2H, Ar-H *ortho* to CO), 8.10 (d, *J* = 8.8 Hz, 2H, Ar-H *ortho* to N=N), 7.99 (d, *J* = 7.6 Hz, 2H, Ar-H *ortho* to N=N), 7.68–7.65 (m, 3H, Ar-H *meta/para*), 2.93 (s, 4H, NHS-CH_2) ppm (Figure S2).

2.4. Synthesis of N-(2-Aminoethyl)-4-(2-phenyldiazenyl)benzamide

This compound has previously been synthesized via a pentafluorophenyl ester intermediate [3]. In this study, we synthesized the target amide via the NHS ester intermediate prepared above.

A solution of *N*-succinimidyl-4-(phenylazo)benzoate (200 mg, 0.62 mmol) in anhydrous THF (10 mL) was added dropwise to a solution of EDA (2 mL) in anhydrous THF (20 mL). The mixture was then stirred overnight at room temperature. The reaction mixture was diluted with DCM (20 mL) and washed twice with aqueous NaOH (30 mL, pH 12). The solvent was removed under reduced pressure, and the resulting residue was dried overnight under vacuum to afford the final product (136.1 mg, 82%). ^{1}H NMR (400 MHz, DMSO-d_6): δ 8.60 (t, *J* = 5.6 Hz, 1H, CONH), 8.07 (d, *J* = 8.5 Hz, 2H, Ar-H *ortho* to amide), 7.96 (d, *J* = 8.5 Hz, 4H, Ar-H *ortho* to N=N), 7.64–7.61 (m, 3H, Ar-H *meta/para*), 3.31 (q, *J* = 6.0 Hz, 2H, -CH_2-NHCO-), 2.71 (t, *J* = 6.5 Hz, 2H, -CH_2-NH_2) ppm (Figure S2).

AzoBAM-2k

^{1}H NMR (400 MHz, DMSO-d_6): δ 8.66 (br s, 1H, CONH), 8.06 (d, *J* = 8.8 Hz, 2H, Ar-H), 7.95 (d, *J* = 8.8 Hz, 2H, Ar-H), 7.65–7.60 (m, 3H, Ar-H), 5.33–5.30 (m, 2H, Oleyl -CH=CH-), 4.10 (t, *J* = 5.2 Hz, 2H, -CH_2-OCO-), 3.51 (br s, 171H, PEG -CH_2-), 2.37 (t, *J* = 7.2 Hz, 2H, -CH_2-CONH-), 1.98 (m, 4H, Oleyl allylic), 1.46–1.23 (m, 22H, Oleyl -CH_2-), 0.85 (t, *J* = 6.8 Hz, 3H, -CH_3).

AzoBAM-4k

^{1}H NMR (400 MHz, DMSO-d_6): δ 8.66 (t, 1H, CONH), 8.05 (d, *J* = 8.8 Hz, 2H, Ar-H), 7.96 (d, *J* = 8.8 Hz, 2H, Ar-H), 7.63–7.61 (m, 3H, Ar-H), 5.33–5.31 (m, 2H, Oleyl -CH=CH-), 4.10 (t, *J* = 4.8 Hz, 2H, -CH_2-OCO-), 3.51 (br s, 358H, PEG -CH_2-), 2.37 (t, *J* = 7.0 Hz, 2H, -CH_2-CONH-), 1.99–1.97 (m, 4H, Oleyl allylic), 1.47–1.23 (m, 26H, Oleyl -CH_2-), 0.85 (t, *J* = 6.9 Hz, 3H, -CH_3).

AzoBAM-8k

^{1}H NMR (400 MHz, DMSO-d_6): δ 8.66 (t, 1H, CONH), 8.05 (d, *J* = 8.7 Hz, 2H, Ar-H), 7.96 (d, *J* = 8.8 Hz, 2H, Ar-H), 7.65–7.61 (m, 3H, Ar-H), 5.33–5.31 (m, 2H, Oleyl -CH=CH-), 4.10 (t, *J* = 4.8 Hz, 2H, -CH_2-OCO-), 3.51 (br s, 746H, PEG -CH_2-), 2.37 (t, *J* = 7.0 Hz, 2H, -CH_2-CONH-), 1.99–1.97 (m, 4H, Oleyl allylic), 1.47–1.24 (m, 26H, Oleyl -CH_2-), 0.85 (t, *J* = 6.9 Hz, 3H, -CH_3).

2.5. Isothermal Titration Calorimetry (ITC)

ITC measurements were performed using a MicroCal PEAQ-ITC (Malvern Panalytical) at 25 °C. AzoBAM variants (2k, 4k, and 8k) were dissolved in PBS and placed in the sample cell. A solution of β-CD was prepared in the same solvent at a concentration of 5 mM and loaded into the injection syringe. The titration protocol consisted of an initial injection of 0.4 μL followed by 18 injections of 2.0 μL with a spacing of 150 s between injections to ensure thermal equilibrium. The stirring speed was maintained at 750 rpm. Data integration and processing were performed using the MicroCal PEAQ-ITC Analysis Software. Due to the complex thermal profiles observed for the AzoBAM-4k variant and the negligible heat release for AzoBAM-2k, curve fitting to a standard binding model was not performed; instead, the integrated enthalpy trends were analyzed qualitatively.

2.6. Cytotoxicity test

K562 cell suspension was aliquoted into a 24-well plate (800 μL/well). The cells were exposed to UV irradiation (365 nm, intensity: 16.6 W/m^2) for varying durations (0, 1, and 5 minutes). Cell viability and proliferation were evaluated immediately (0 h) and 24 hours after irradiation. For the assay, cells were washed twice with PBS and then stained with Calcein-AM and Propidium Iodide (PI) diluted 1:500 in PBS to label live (green) and dead (red) cells, respectively. Cell viability and cell density were quantified based on the fluorescence signals.

3. Supplementary Figures

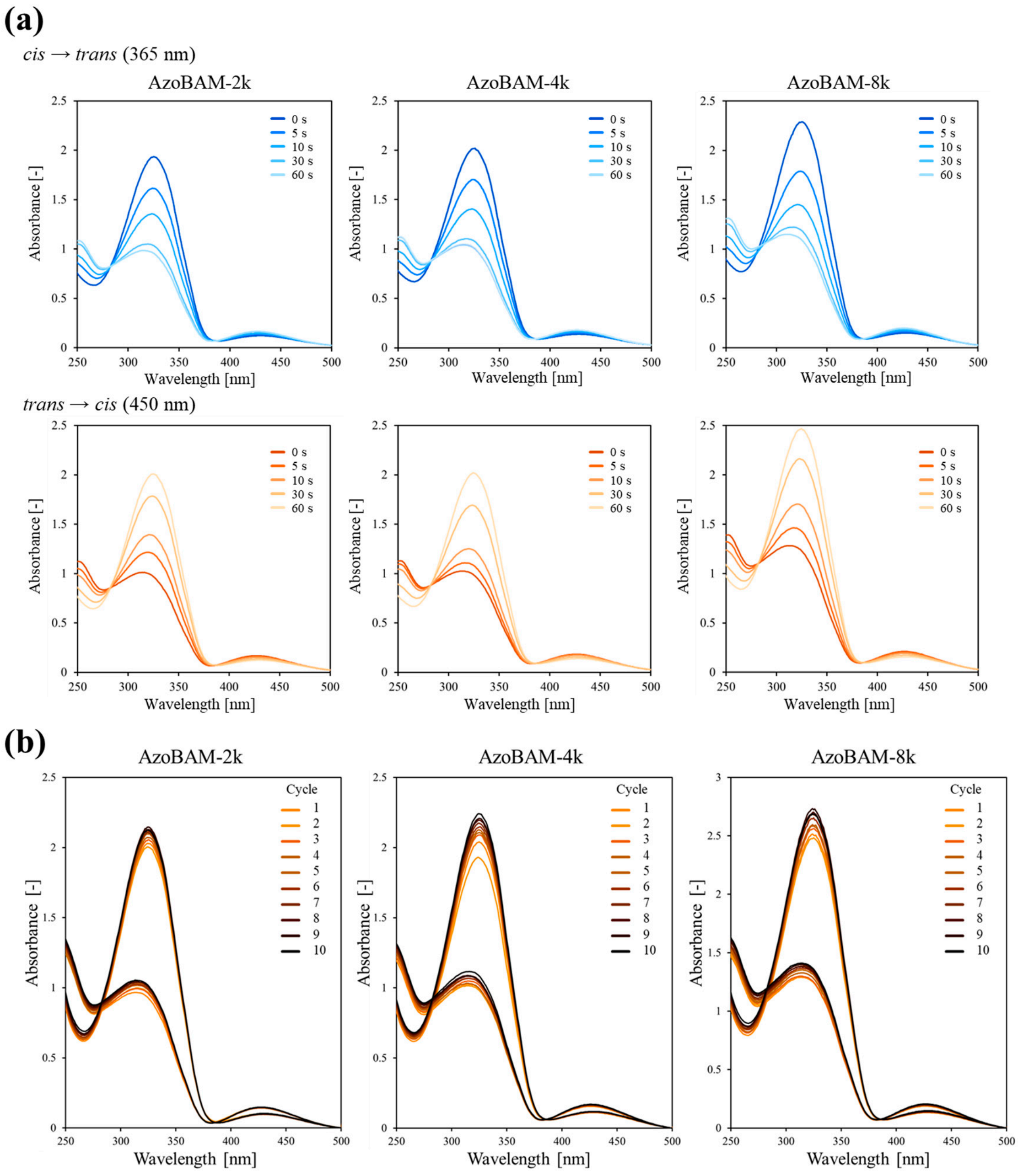

Figure S1. (a) UV-vis absorbance spectra showing the photoisomerization of AzoBAM. Top: *trans*-to-*cis* transition; Bottom: *cis*-to-*trans* transition. The spectra were recorded after irradiation for 0, 5, 10, 30, and 60 s. (b)Reversibility of photoisomerization over 20 cycles for AzoBAM-2k, 4k, and 8k (from left to right). The *trans*-to-*cis* transition was induced by 365 nm irradiation (16.6 W/m^2), and the cis-to-trans transition by 450 nm irradiation (157 W/m^2). Spectra were recorded after 60 s of irradiation for each step.

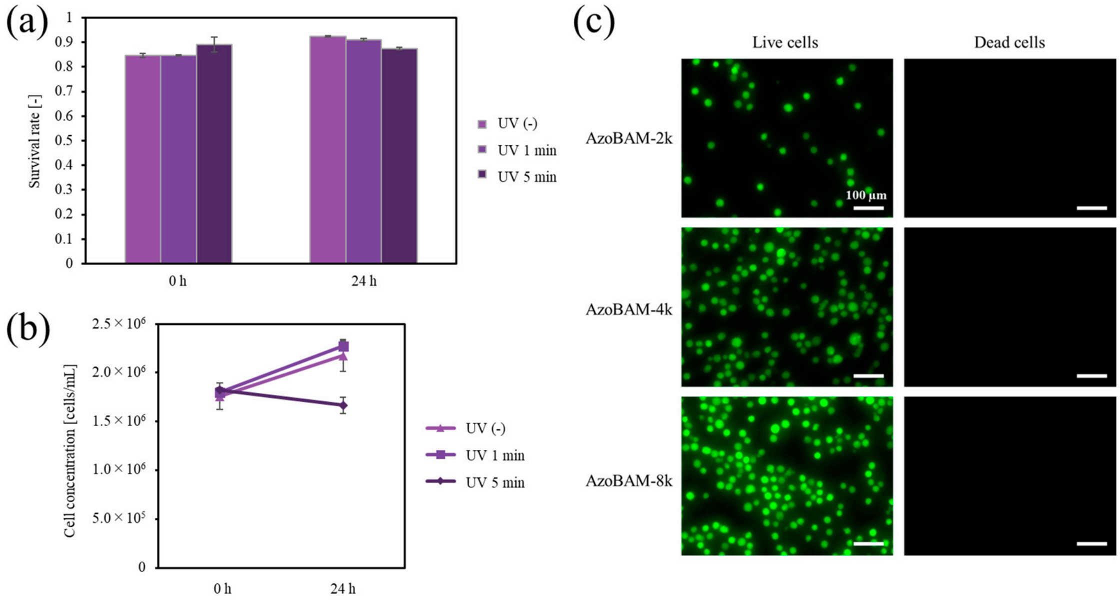

Figure S2. Evaluation of UV-induced cytotoxicity. (a) Cell survival rate and (b) cell density measured at 0 and 24 h after UV irradiation. All conditions (0, 1, and 5 min irradiation) showed comparable survival rates. However, while the 1 min irradiation group showed cell growth similar to the non-irradiated control (0 min), the 5 min irradiation group exhibited inhibited proliferation after 24 h. (c) Assessment of acute membrane damage in preliminary experiments. Fluorescence images of cells following AzoBAM treatment. Left panels: Live cells stained with Calcein-AM (green fluorescence). Right panels: Dead cells stained with Propidium Iodide (PI) (red fluorescence). Note the absence of significant PI signals, indicating negligible acute membrane damage under the applied conditions. Scale bars; 100 µm.

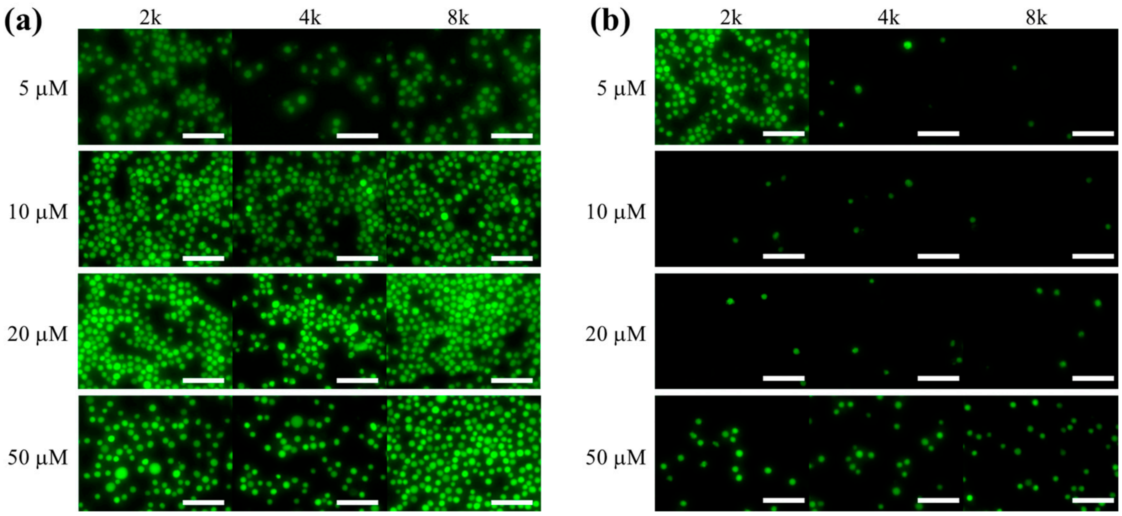

Figure S3. Fluorescence microscopy images of cell adhesion for all AzoBAM variants (2k, 4k, 8k) at various concentrations (5, 10, 20, and 50 μM). (a) Adhered cells before UV irradiation. (b) Remaining cells after irradiation with 365 nm light for 1 min, followed by washing. Green fluorescence indicates live cells stained with Calcein-AM. Scale bars; 50 μm.

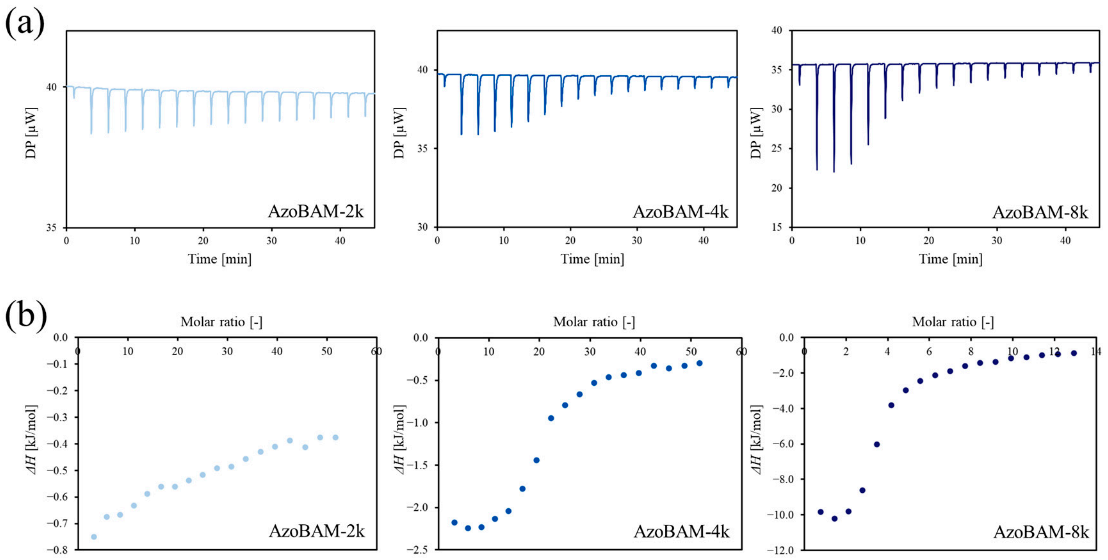

Figure S4. Isothermal Titration Calorimetry profiles for the interaction between β-CD and AzoBAM variants. (a) Raw thermograms (differential power vs. time) and (b) integrated heat plots (enthalpy change vs. molar ratio) for AzoBAM variants (2k, 4k, 8k) titrated into the β-CD solution at 25°C. Note that AzoBAM-2k showed negligible heat changes, indicating a lack of specific binding due to stable micelle formation. In contrast, AzoBAM-8k exhibited prominent exothermic heat, consistent with the unhindered binding of free polymer chains. AzoBAM-4k displayed reduced exothermic heat compared to the 8k variant, suggesting a competition between host–guest binding and de-aggregation processes (energetic penalty). Curve fitting was not performed due to the complex thermal profiles.

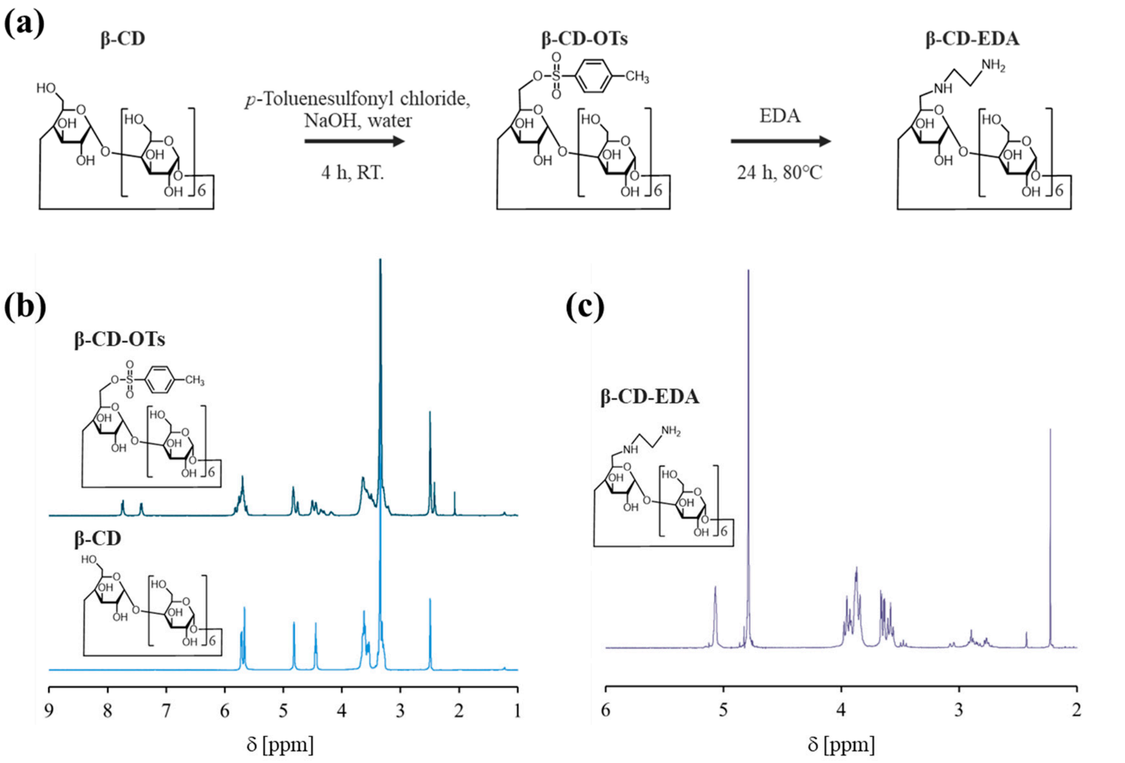

Figure S5. Synthesis and NMR characterization of β-cyclodextrin (β-CD) derivatives. (a) Synthetic scheme of mono-6-(2-aminoethyl)amino-6-deoxy-β-CD (β-CD-EDA). (b) ^{1}H NMR spectra of β-CD and mono-6-tosyl-β-CD (β-CD-OTs) in DMSO-d_6. (c) ^{1}H NMR spectrum of β-CD-EDA in D_2O.

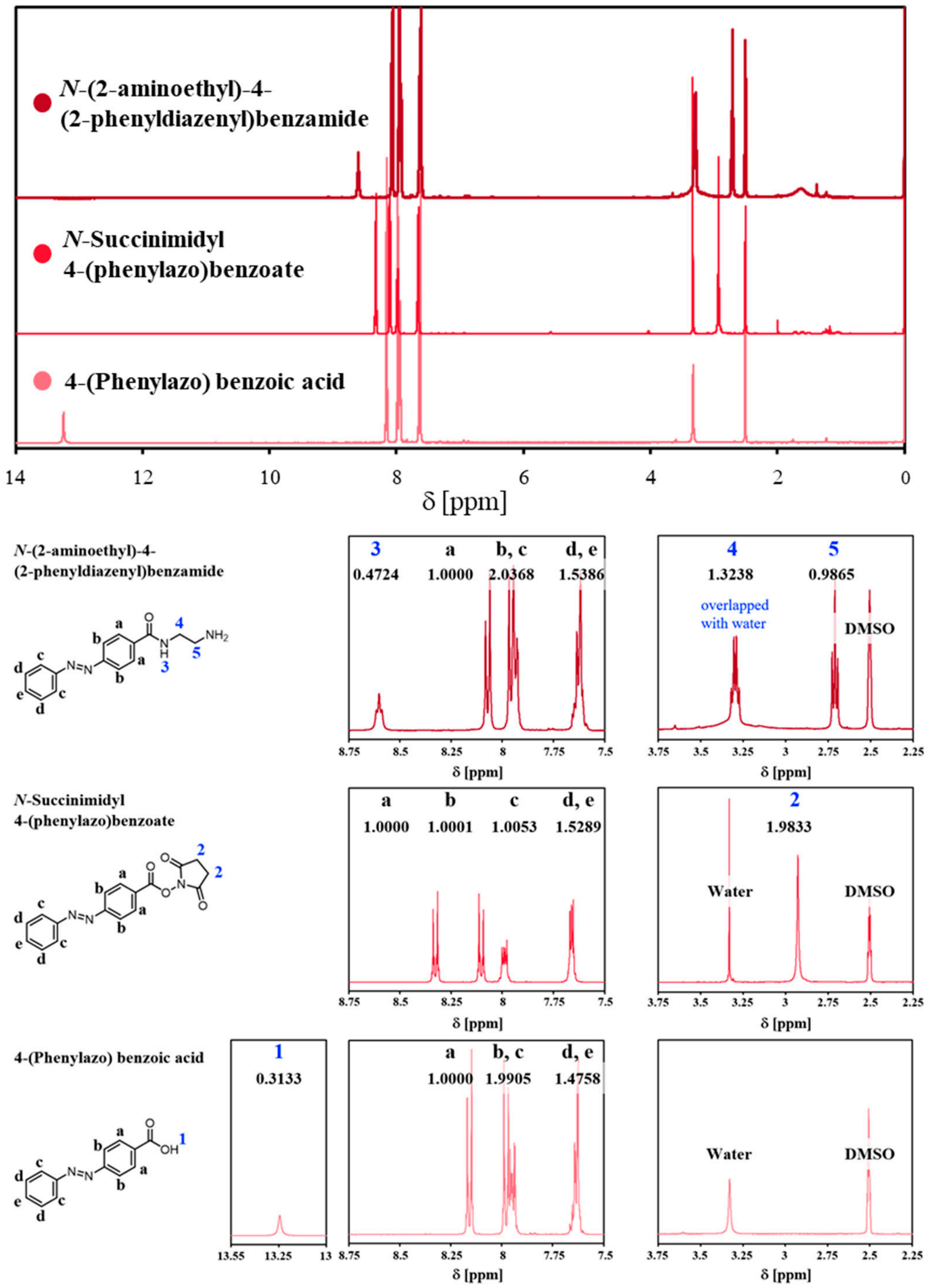

Figure S6. Expanded ^{1}H NMR spectra of 4-(phenylazo)benzoic acid, *N*-succinimidyl 4-(phenylazo)benzoate, and *N*-(2-aminoethyl)-4-(2-phenyldiazenyl)benzamide. Integration values are shown in the graphs. Comparison of the spectra reveals that the signal at 8.14 ppm in the NHS ester corresponds to the protons *ortho* to the ester group, which shifted downfield due to the electron-withdrawing effect. The integration values (2H) confirm the stoichiometric consistency.

References

[1] S. Payamifar & A. P. Marjani, A new β-cyclodextrin-based nickel as green and water-soluble supramolecular catalysts for aqueous Suzuki reaction, scientific reports, 2023, 13, 21279, DOI: 10.1038/s41598-023-48603-6.

[2] S. Keiper, J. S. Vyle, Reversible Photocontrol of Deoxyribozyme-Catalyzed RNA Cleavage under Multiple-Turnover Conditions, *Angew. Chem. Int. Ed.*, 2006, 45, 20, 3306-3309, DOI: 10.1002/anie.200600164.

[3] F. D. Jochum, P. Theato, Temperature and light sensitive copolymers containing azobenzene moieties prepared via a polymer analogous reaction, *Polymer*, 2009, 50, 14, 3079-3085, DOI: 10.1016/j.polymer.2009.05.041.
